# Investigating the Influence of Morphine and Cocaine on the Mesolimbic Pathway Using a Novel Microimaging Platform

**DOI:** 10.3390/ijms242216303

**Published:** 2023-11-14

**Authors:** Austin Ganaway, Kousuke Tatsuta, Virgil Christian Garcia Castillo, Ryoma Okada, Yoshinori Sunaga, Yasumi Ohta, Jun Ohta, Masahiro Ohsawa, Metin Akay, Yasemin M. Akay

**Affiliations:** 1 Biomedical Engineering Department, University of Houston, 3517 Cullen Blvd, Houston, TX 77204, USA; amganaway@uh.edu (A.G.); makay@uh.edu (M.A.); 2 Department of Neuropharmacology, Faculty of Pharmaceutical Sciences, Nagoya City University, Nagoya 467-8601, Japan; tatsutakousuke@gmail.com (K.T.); oosawa.masahiro.bg@teikyo-u.ac.jp (M.O.); 3 Division of Materials Science, Graduate School of Science and Technology, Nara Institute of Science and Technology, Ikoma 630-0101, Japan; castillo.virgil_christian.cr3@ms.naist.jp (V.C.G.C.); okada.ryoma.on9@ms.naist.jp (R.O.); sunaga.yoshinori@ms.naist.jp (Y.S.); ohtay@ms.naist.jp (Y.O.); ohta@ms.naist.jp (J.O.)

**Keywords:** morphine, cocaine, dopamine, VTA, NAc, CMOS imaging, dLight AAV, fluorescence imaging

## Abstract

Dopamine (DA)’s relationship with addiction is complex, and the related pathways in the mesocorticolimbic system are used to deliver DA, regulating both behavioral and perceptual actions. Specifically, the mesolimbic pathway connecting the ventral tegmental area (VTA) and the nucleus accumbens (NAc) is crucial in regulating memory, emotion, motivation, and behavior due to its responsibility to modulate dopamine. To better investigate the relationship between DA and addiction, more advanced mapping methods are necessary to monitor its production and propagation accurately and efficiently. In this study, we incorporate dLight1.2 adeno-associated virus (AAV) into our latest CMOS (complementary metal-oxide semiconductor) imaging platform to investigate the effects of two pharmacological substances, morphine and cocaine, in the NAc using adult mice. By implanting our self-fabricated CMOS imaging device into the deep brain, fluorescence imaging of the NAc using the dLight1.2 AAV allows for the visualization of DA molecules delivered from the VTA in real time. Our results suggest that changes in extracellular DA can be observed with this adapted system, showing potential for new applications and methods for approaching addiction studies. Additionally, we can identify the unique characteristic trend of DA release for both morphine and cocaine, further validating the underlying biochemical mechanisms used to modulate dopaminergic activation.

## 1. Introduction

Addiction and dopamine (DA) intertwine at a synaptic level, as the dopaminergic pathways are heavily implicated with substance abuse [1,2,3]. Key neurotransmitters modulate these pathways, regulating both behavioral and perceptual actions. While many neurochemicals play regulatory roles in addiction mechanisms, DA is unique in its function [4,5]. The mesolimbic pathway, a dopaminergic projection primarily from the ventral tegmental area (VTA) to the nucleus accumbens (NAc), is crucial in controlling the behavioral response to both emotion and interpretation of pain and pleasure [4,6]. The primary role of the VTA relates to reward and addiction; positive stimuli from addictive sources, such as food or drugs, cause the activation of dopaminergic neurons, inciting the production and projected release of DA [7]. Receiving the DA, the NAc assists in regulating memory, emotion, motivation, and behavior [7,8]. Therefore, investigating prescriptive and illicit drugs is necessary to characterize further DA’s role in regulating addiction [9]. Morphine is an opioid used for pain management and commonly distributed by prescribers. Since it is a highly addictive substance, those for whom a prescription is necessary must accept the risk that they may become dependent on the substance, leading to substance abuse. An entirely illicit stimulant, cocaine is pervasive in terms of illegal drug activity. Due to its potency, it is incredibly addictive, and overdose cases can quickly morph into life-threatening situations [10]. By introducing our complementary metal-oxide semiconductor (CMOS) imaging platform to the NAc, we are able to visualize the release of the projected DA using fluorescence, and we can compare the resultant variation in DA activity between morphine and cocaine administration.

Crucially, both morphine and cocaine modulate the dopaminergic activity of the mesolimbic pathway, but the fundamental mechanisms of the drugs differ from each other, making them model substances for investigating our unique fluorescence imaging platform. Morphine attaches to the opioid receptors of the GABAergic interneuron, causing the attenuation of GABA production. Being that GABA directly regulates the production of DA, inhibition of GABAergic neurons leads to the uninhibited production of DA in the VTA. On the other hand, cocaine modulates DA release by blocking the dopamine transporter protein (DAT) of the dopaminergic neuron, which enables the reuptake of DA from the extracellular space. By inhibiting the reuptake of DA from the synaptic cleft, extended stimulation of the NAc occurs. Potentially, our experimental platform may detect the difference in stimulation using fluorescence imaging. Therefore, investigating the relationship between these drugs and the mesocorticolimbic system is crucial to better understand addiction.

Adaptive techniques for visualization of the neural network are paramount to better understanding the intricacies of the mesolimbic pathway and addiction. Many methods exist for analysis, but few can overcome the omnipresent obstacles that entail introducing freely moving experimental design, especially on smaller animals such as mice. Fiberoptic photometry and fluorescence microscopy are suitable methods for many experimental studies. Still, the constraints often impede detailed neural network mapping using freely moving conditions, which is necessary to replicate natural dopaminergic excitation. While these methods excel for in vivo applications using a restrained, sedated subject, when introducing free movement, the optical fiber setup is rigid and fragile and potentially limits the range of movement provided to the animal [11].

Studies involving a GRIN lens can bypass specificity issues by focusing on a particular area of neural tissue on a micrometer scale. Nevertheless, the fragile and bulky hardware required, paired with difficult implantation and post-operative care, leads to many new issues, hindering feasibility and weakening visibility due to poor control of light scattering [12]. Additionally, these methods provide a horizontal viewing plane, a superficial viewing angle that leaves out potentially crucial interactions. An alternative approach that demonstrates promise and facilitates freely moving conditions is the microdialysis of the mesolimbic pathway. The ability to quantify DA output in real time is invaluable, and the microdialysis probe can be accurately implanted into specific areas, assisting in the issue of spatial resolution. Despite these improvements, the temporal resolution is inferior because the low flow rate has an inverse relationship corresponding to accuracy, with samples collected every 15–30 min [13]. Leaving substantial gaps between each sample inhibits the ability to understand the dopaminergic activity comprehensively.

Extracellular recording focuses on the electrical signal of a specific neuron, such as single unit recording, or the electrical transmissions of a general area, such as local field potential recordings. Crucially, electrodes introduce complex, fragile structures to the subject’s skull. While microdialysis allows for analyzing the DA neurotransmitter in a specific brain region, its weak temporal resolution introduces a different slew of difficultly managed variables. By subverting traditional deep brain recording methods such as extracellular recording and microdialysis, we can localize and identify the presence of specific neurotransmitter activity with excellent temporal and spatial resolution. We also introduce the ability to monitor specific types of neurons en masse selectively, such as dopaminergic neurons, or visualize even particular neurotransmitters, such as DA. 

To achieve detailed monitoring of drug-induced dopaminergic activity concerning both spatial and temporal resolution while maintaining freely moving integrity within the mesocorticolimbic system, our previous research introduced a novel CMOS microimaging device with a customized micro-CMOS sensor accompanied by micro-LEDs on a flexible printed circuit (FPC) substrate [14,15,16]. The device is constructed of polyamide, coated with Parylene-C for biocompatibility, and is implanted directly into the brains of both rats and mice. Freely moving experimentation is facilitated by its light weight, flexibility, and ease of implantation. Additionally, our CMOS device allows for a vertical view of the brain, which is notoriously difficult to achieve with conventional methods.

Our previous research demonstrated efficacy in investigating the excitation of dopaminergic neurons in the VTA in both mice and rats. Initially, we observed fluorescence activity enabled by the GCaMP6 adeno-associated virus (AAV) using freely moving conditions of adult rats. By using an AAV, we can introduce genetic changes to neuronal tissue which enable the visualization of fluorescence due to fluctuations in calcium. The GCaMP6 AAV is an engineered genetically encoded calcium indicator (GECI) that incorporates green fluorescent protein (GFP) and calmodulin, which is a protein that binds to calcium. The administration of varying nicotine dosages resulted in fluctuations in fluorescence intensity, with the change in fluorescence intensity (*ΔF*) being directly proportional to the strength of the nicotine dosage [14]. We then verified the relationship between the change in fluorescence intensity and excitation of dopaminergic neurons by introducing our CMOS imaging platform to adult mice in tandem with an analysis of microdialysis perfusate obtained from the NAc [15]. Finally, we recently adapted the CMOS imaging platform to observe serotonin activation of the DRN following pain stimulation incorporated with microdialysis [16].

**Figure 1:**
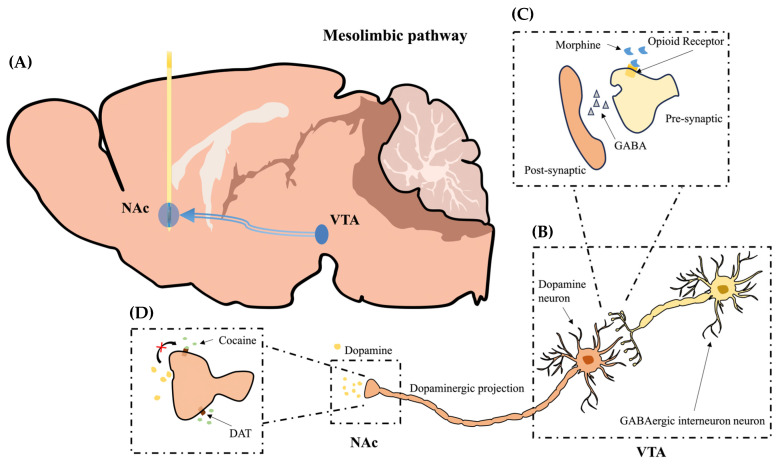
Visualization of the relationship between the mesolimbic pathway and the adaptable CMOS imaging device implantation. (**A**) The CMOS imaging device is implanted into the NAc, facing medially, obtaining a vertically oriented viewing plane encapsulating the length of the NAc. The dLight1.2 virus expressed in the NAc allows for fluorescent graphical representation of projected DA molecules. (**B**) Dopaminergic projections extend from the VTA to the NAc. Following drug-induced stimulation of the dopaminergic neuronal hub located in the VTA, activity of DA neurons is modulated, and they dump DA molecules into the NAc via the mesolimbic pathway. (**C**) Upon morphine administration, GABAergic interneurons are inhibited due to the blocking of the opioid receptor on the pre-synaptic terminal. Due to decreased GABA release, DA neurons are unattenuated for an extended period, leading to increased DA release to the NAc. (**D**) Following cocaine administration, the dopamine transporter (DAT) protein is blocked. The reuptake of DA by DAT from the extracellular space is inhibited, which is a crucial part of terminal DA signal transmission between neurons.

In this study, we investigate the influence of morphine and cocaine on the mesolimbic pathway by introducing the dLight1.2 AAV to the NAc of adult mice and monitor the DA release using our adaptable microimaging platform consisting of a micro-CMOS sensor and micro-LEDs during freely moving experimentation to visualize the effects of drug-induced DA release (Figure 1). While previous studies monitored the activation of dopaminergic neurons, they could not visualize the release of DA itself to projected areas pertinent to addiction. By applying our experimental system to the NAc and visualizing the release of the DA neurotransmitter, confirmation of previous microdialysis results can be validated while simultaneously demonstrating improved spatial and temporal resolution for real-time neurotransmitter monitoring. Additionally, analysis of DA release trends based on morphine or cocaine administration can further verify previous findings regarding the biochemical mechanisms responsible for dopaminergic modulation in the mesocorticolimbic system. 

## 2. Results

### 2.1. Morphine-Induced Dopaminergic Stimulation Increased DA Release in NAc

The fluorescence activity within the NAc of adult C57BL/6 wild-type mice was investigated to determine the efficacy of our CMOS imaging experimental platform regarding the identification and visualization of the release of DA molecules and to identify a positive correlation between the administration of pharmacological stimulation and the activation of the mesolimbic pathway in real time. dLight1.2 AAV was allowed two weeks to express within the NAc adequately. Each device was implanted in the brain’s right hemisphere, and a singular experimental protocol was followed for every experiment. Following the experimental setup, mice were left undisturbed for 2 h to establish a fluorescence baseline to act as a control. Mice were administered a dosage of 3.0 mg/kg of morphine via subcutaneous injection to induce dopaminergic stimulation, meaning stimulation of the mesolimbic pathway.

**Figure 2:**
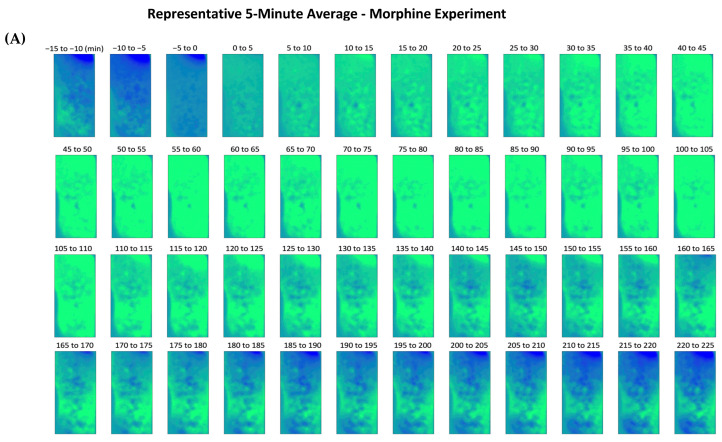

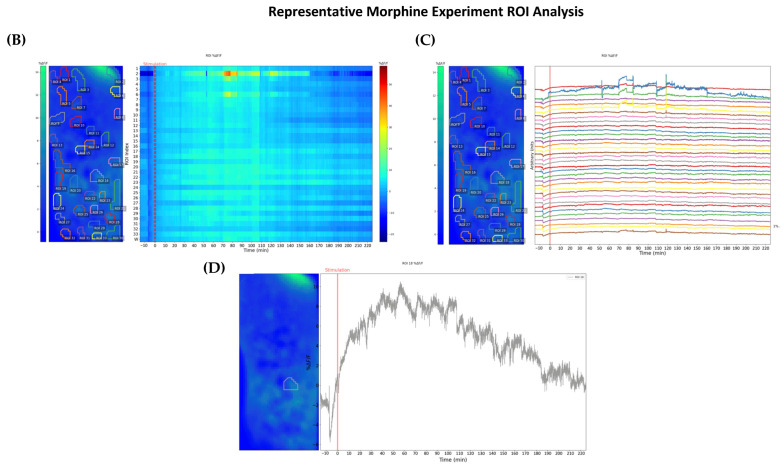
In vivo fluorescence imaging of the NAc demonstrated with a representative adult mouse and fluorescence intensity changes (*ΔF/F*_0_) in the NAc following a subcutaneous morphine injection. (**A**) Averaged 5-min intervals of fluorescent imaging are presented for the relevant drug-induced excitation timeframe of the mouse during the experiment. The first three averages occur before subcutaneous injection of morphine, and the following averages demonstrate the increase in projected extracellular DA following stimulation. The flexible CMOS imaging device allowed for freely moving experimentation throughout the entire experiment. (**B**) Fluorescence intensity, depicted using a color scale, correlates to extracellular DA concentration in the NAc directly. On the left, the average change in fluorescence from 15 min pre-administration to 225 min post-administration is depicted, and regions of interest (RIOs) are identified using analysis software. On the right, a heatmap of the change in fluorescence intensity over time is shown, with each line corresponding to its respective ROI. Each number correlates to a specific ROI, and W refers to the whole image. (**C**) Graphical representation of each ROI throughout the experiment shows each ROI’s change in intensity. A different color represents each ROI. (**D**) Graphical representation of a single ROI depicting the change in fluorescence intensity throughout the experiment.

The fluorescence activity average over 5-min intervals was obtained following analysis of the data from morphine experimentation, as shown using a representative mouse in Figure 2A. Moderate, stable fluorescence was observed in the minutes before the drug administration. The pharmacologically induced DA release was characterized by a steady incline in fluorescence intensity, with increased intensity occurring between the 60- and 115-min marks. As the effect of the morphine injection began to wane, the fluorescence intensity decreased throughout the image, dropping below baseline. Correlating fluorescence intensity to the presence of extracellular DA, distinct regions of the images captured by the CMOS device reveal an increase in extracellular DA. 

**Figure 3:**
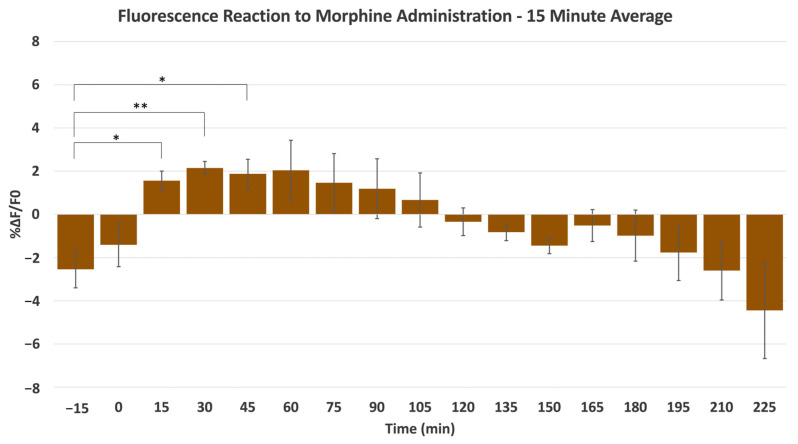
The average fluorescence intensity changes (*ΔF/F*_0_) in the NAc from a subcutaneous morphine injection, with each interval averaged over a 15-min period and each animal receiving a single injection (*n* = 3). A significant difference was observed between the pre-15-min control and the 15-, 30-, and 45-min marks. ** denotes *p* < 0.01, ±SD. * denotes *p* < 0.05, ±SD.

To better qualify the change in fluorescence intensity, regions of interest (ROIs) were selected, taking advantage of a custom-built algorithm rooted in adaptive binarization. Using this technique, the program analyzes the features of every frame, detecting even minute changes in fluorescence intensity. Analysis of each ROI is derived from 15 min pre-administration to 225 min post-administration. In Figure 2B–D, analysis results from the representative morphine experiment are presented, further illustrating the increase in *ΔF/F*_0_ percentage depicted in Figure 2A. Figure 2B depicts a heatmap corresponding to the 33 ROIs and the whole image (W) identified by the program, with each ROI being provided with a corresponding number and horizontal fluorescence intensity bar. The heatmap visually portrays the duration of the drug’s effect and highlights the drug release and response characteristics. Linear representation of the change in fluorescence intensity for each respective ROI is depicted in Figure 2C, following the subcutaneous injection of morphine. An increase in intensity is observed in each ROI, with the signal peaking near the 60-min mark. To observe the change in *ΔF/F*_0_ percentage more clearly, Figure 2D introduces a representative ROI illustrating the trend observed from morphine-induced stimulation. As witnessed in the previous figures, the extracellular DA concentration reaches its peak near the 60-min mark. Figure 2 highlights the characteristic trend presented following morphine stimulation, denoted by a gradual spike and a slow decline over time. Figure 3 shows the average fluorescence intensity of all of the morphine experiments, with each time point averaging over 15 min. We establish a significant increase in fluorescence intensity at multiple points following morphine administration during experimentation compared to the pre-15-min mark average. The peak intensity falls between 30 and 60 min, and a gradual increase and decrease in intensity can characterize the curve. 

### 2.2. Cocaine-Induced Dopaminergic Stimulation Modulates DA Levels in NAc

**Figure 4:**
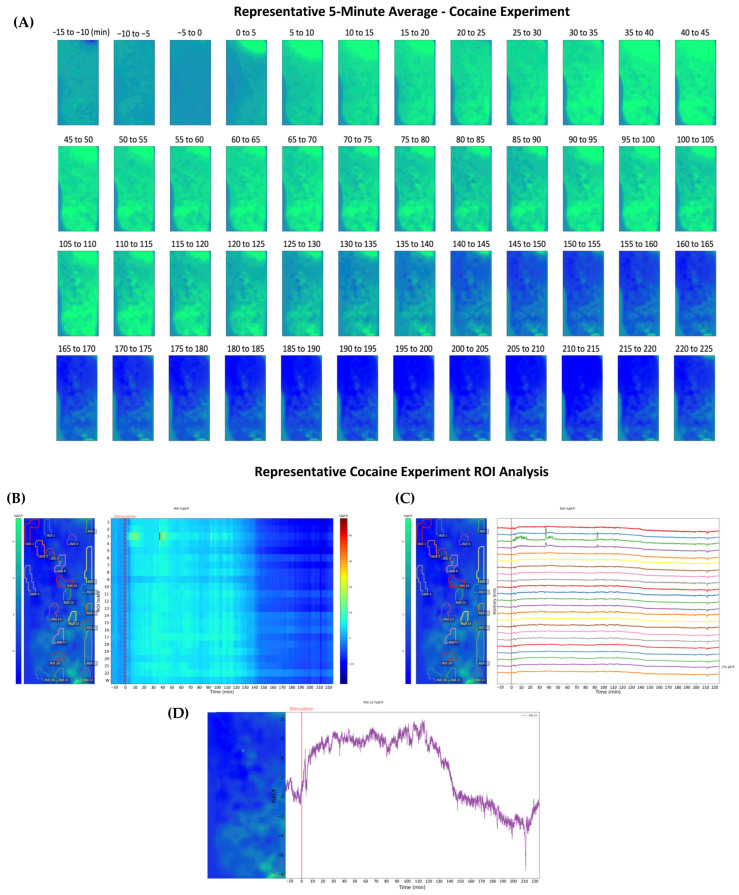
In vivo fluorescence imaging of the NAc demonstrated with a representative adult mouse and fluorescence intensity changes (*ΔF/F*_0_) in the NAc following a subcutaneous cocaine injection. (**A**) Averaged 5-min intervals of fluorescent imaging are presented for the relevant drug-induced excitation timeframe of the mouse during the experiment. The first three averages occur before subcutaneous injection of cocaine, and the following averages demonstrate the modulation of projected extracellular DA following stimulation. The flexible CMOS imaging device allowed for freely moving experimentation throughout the entire experiment. (**B**) Fluorescence intensity, depicted using a color scale, has been determined to correlate to extracellular DA concentration in the NAc directly. On the left, the average change in fluorescence from 15 min pre-administration to 225 min post-administration is depicted, and regions of interest (RIOs) are identified using analysis software. On the right, a heatmap of the change in fluorescence intensity over time is shown, with each line corresponding to its respective ROI. Each number correlates to a specific ROI, and *W* refers to the whole image. (**C**) Graphical representation of each ROI throughout the experiment shows each ROI’s change in intensity. A different color represents each ROI. (**D**) Graphical representation of a single ROI depicting the change in fluorescence intensity throughout the experiment.

Following the same experimental protocol used to induce and measure opioid activation of the NAc, we investigated cocaine-induced dopaminergic modulation to compare the activation trend to morphine. At the start of experimentation, animals were allowed 2 h before subcutaneous injection to stabilize and adapt to the freely moving enclosure. The mice were then administered a 10 mg/kg bolus of cocaine subcutaneously, and fluorescence was recorded. Fluorescence averaging over 5-min intervals was again processed for the duration of the experiment, as shown by the representative animal in Figure 4A. A decrease in fluorescence due to handling noise signifies the injection time at the 0–5-min image. An immediate increase in fluorescence is observed following the administration of cocaine, with the intensity reaching its peak at the 40–45-min mark. A more sudden drop in fluorescence intensity is witnessed relative to the prior opioid administration experiments. 

**Figure 5:**
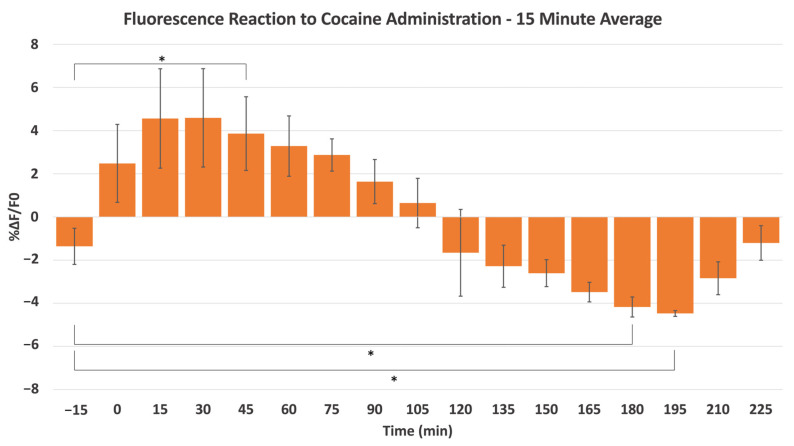
The average fluorescence intensity changes (*ΔF/F*_0_) in the NAc from a subcutaneous cocaine injection, with each interval averaged over a 15-min period and each animal receiving a single injection (*n* = 3). A significant difference was observed between the pre-15-min control, the 45-min mark, the 180-min mark, and the 195-min mark. * Denotes *p* < 0.05, ±SD.

Additional signal processing was performed to characterize the DA response after cocaine exposure. Figure 4B,C serves to visualize the suggested dopaminergic response of the mesolimbic system. As with morphine, Figure 4B provides a heatmap highlighting specific ROIs identified by sophisticated analysis software, which uses the detection of small changes in fluorescence intensity to encapsulate areas of heightened activity visually. Due to the rapid response and stark decrease in *ΔF/F*_0_ percentage following stimulation, the effects of the stimulant are clearly defined, with an almost immediate reaction occurring after the injection time, an unambiguous drop witnessed near the 140-min mark. In a more linear form, Figure 4C shows ROIs following a similar trend of immediate response and sudden drop-off in fluorescence intensity. Finally, a close-up of ROI 22 provides a detailed visualization of the response pattern initiated by cocaine administration, with a substantial decrease in *ΔF/F*_0_ percentage occurring between 120 and 150 min in Figure 4D. Figure 5 shows the average of the three individual experiments before and after cocaine administration, with each time point representing the average over a 15-min period. A significant increase was observed following cocaine administration compared to the pre-15-min control. Additionally, the DA release trend can be characterized as rapid and intense, with a peak between the 15- and 45-min marks and a faster return to baseline than its morphine counterpart. 

**Figure 6:**
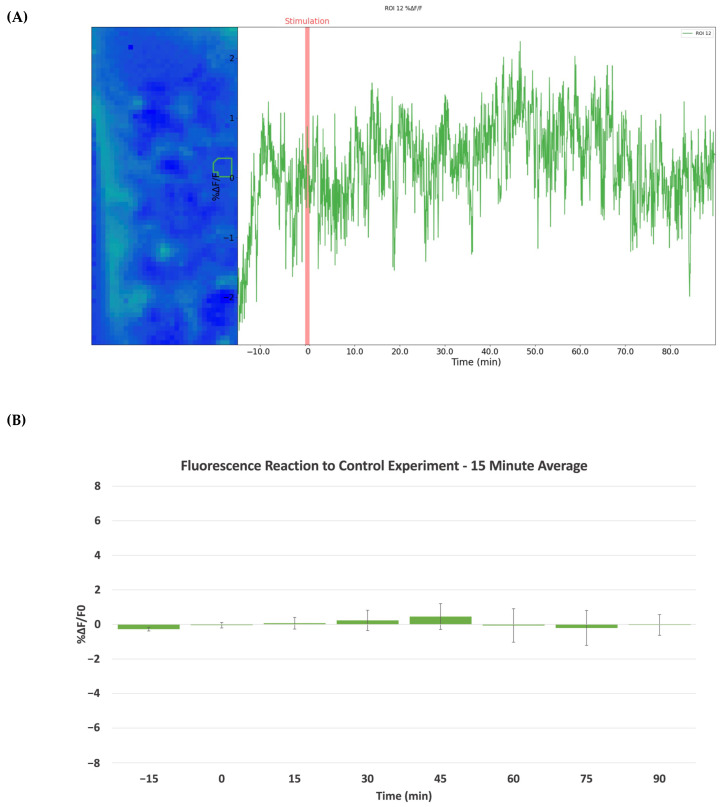
Results of control experiment visualizing fluorescence activity of the NAc of mice before and after subcutaneous injection of saline. (**A**) A representative animal is shown to reflect the fluorescence intensity changes (*ΔF/F*_0_) following control injection. (**B**) The average *ΔF/F*_0_ in the NAc from control experiments, with each interval averaged over a 15-min period. Each animal received a single injection (*n* = 3). No significant difference was observed between any time points.

To validate our findings regarding both morphine and cocaine stimulation, fluorescence imaging of the NAc of control animals injected with saline was performed. The experiment was conducted over 105 min, with 15 min before the subcutaneous injection serving to establish a baseline and 90 min post-injection to observe the change in fluorescence intensity. Figure 6 depicts the experimental results of the control group. Figure 6A provides the change in fluorescence intensity of the NAc of a representative animal. The change in intensity is negligible, with little variation throughout the duration of the experiment. Figure 6B shows the collective average change in fluorescence intensity during the control experiments (*n* = 3). Each time point is an average of the respective 15-min interval, and the graph is scaled to that of its morphine and cocaine counterparts. No significant difference was noted between any of the time points, and the change in fluorescence intensity is considerably less than that demonstrated by the cocaine and morphine animals.

### 2.3. Confirmation of Virus Expression and Implanted CMOS Imaging Device Position

**Figure 7:**
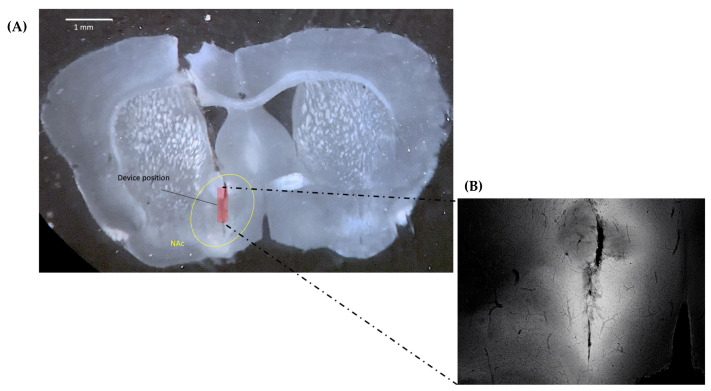
Post-experimental analysis of brain tissue was performed to verify the location of the CMOS device implantation and the success of the dLight1.2 AAV injection and expression. (**A**) A bright field image of a representative mouse coronal brain slice following experimentation using an implanted CMOS imaging device. The NAc and device implantation locations are noted. (**B**) Fluorescence imaging of the brain slice showing fluorescence from dLight1.2 expression surrounding the implantation site in the NAc.

To validate that appropriate dLight1.2 AVV expression occurred following the virus injection surgery and to ensure proper implantation location, perfusion fixation was performed as a terminal procedure after final experimentation for each animal, during which 1× phosphate-buffered saline (PBS) was pushed through the cardiovascular system of the mouse to remove excess blood and fluids. 4% paraformaldehyde was propagated throughout the animal to fixate the brain tissue, preparing it for slicing using a vibratome. We used fluorescence microscopy to visualize the location of the implantation, which can be observed in Figure 7A. The target location was between −4.3–4.6 mm in the dorsoventral direction, with the sensor facing medially. The implantation location suggests the device was implanted correctly, with the sensor positioned appropriately to visualize the NAc. To ensure that the change in fluorescence intensity is proportional to the extracellular concentration of DA in the NAc, we applied the appropriate activation wavelength to the brain slice to encourage dLight1.2 fluorescence. The residual fluorescence observed around the implantation site allows us to infer the successful injection and expression of the virus, as seen in Figure 7B. Additionally, the observed change in fluorescence activity suggests alterations in extracellular DA delivered by the mesolimbic pathway in the NAc. 

## 3. Discussion

### 3.1. dLight1.2 AAV Advancement with CMOS Imaging Device

Dopaminergic projections of the mesocorticolimbic system are complex in their structure and function, requiring the implementation of novel imaging platforms designed for flexibility and efficiency to examine the brain’s neurochemical processes. Fluorescence imaging is a multifaceted tool in many fields of research. It proves exceptionally useful for neural mapping and imaging due to a wide range of equipment applicable to many AAVs and an incredible array of transgenic animal models. In this study, we apply our modified fluorescence imaging platform to adult mice to investigate morphine’s influence on the mesolimbic pathway. By introducing the dLight1.2 AAV into the NAc, we can visualize the release of the DA molecule with notable spatial and temporal resolution. Our imaging results indicate that we can accurately identify the neural stimulation incited by the administration of morphine and cocaine by referencing the change in fluorescence intensity registered by the implanted CMOS device to the amount of DA released by the dopaminergic projections emanating from the VTA. Expression stability is another critical reason for adapting our experimental platform to neurotransmitter imaging. The dLight1.2 AAV and other genetically encoded markers allow for the consistent stability of expression, enabling us to capture images for any time after the initial two-week incubation period. Our system can take advantage of dLight1.2′s fluorescence properties using its flexible and easily maintainable system platform.

In vivo calcium imaging is a pivotal cornerstone of modern medical engineering, becoming nearly ubiquitous in many fields of research and medical application. In previous studies, we achieved calcium imaging of crucial deep brain locations such as the VTA by taking advantage of foundational fluorescence properties, such as GCaMP’s ability to identify the activity of dopaminergic neurons [14,15]. When a dopaminergic neuron becomes excited, the intracellular calcium concentration increases as calcium floods into the cell. Because GCaMP is a GECI, it can undergo conformational change when it binds to intracellular calcium and fluoresces [17]. Using this method, we have identified the activity of dopaminergic neurons using fluorescence deep brain imaging. Considering the strong efficacy demonstrated by the CMOS imaging platform for tracking subtle changes in neurobiological activity, an investigation into more advanced applications may shed light on subtle changes in neurotransmitter signaling.

By shifting our observation from the intracellular calcium concentration of the dopaminergic cell to the dopamine neurotransmitter itself, we can visualize the release of DA in real time as it reaches its destination. The experimental platform is enabled by the dLight1.2 AAV’s intensity-based design. Foundationally, dLight1.2 relies on the direct coupling of the DA-induced conformational change in DA receptors to the fluorescence intensity of circularly permuted GFP [18]. In doing so, reliable intensity via fluorescence relative to the amount of DA present can be visualized using deep brain imagining, recorded, and processed by our CMOS imaging platform. Since the accumulation and dissipation of the DA molecule itself are being observed, it is vital to interpret the change in fluorescence activity over extended periods. A neuron’s action potential firing occurs faster than the release and reuptake of DA from the extracellular space of the NAc. Therefore, we observed the neuronal fluorescence activity of each mouse over 225 min following injection.

### 3.2. CMOS Imaging Applications

Previously, we performed studies using our CMOS imaging platform using rats and mice to map the dopaminergic addiction-related pathways. We applied our novel CMOS imaging platform to characterize the effects of nicotine on DA release within the subsections of the VTA [14]. Adult female Sprague-Dawley rats injected with GCaMP6 adeno-associated virus underwent experimentation with freely moving conditions during which different nicotine concentrations were injected intraperitoneally (IP). The *ΔF*, defined as the difference between the fluorescence activity (*F*) and the baseline fluorescence activity (*F*_0_), was assessed using specific regions of interest (ROI). For each ROI, the fluorescence intensity increased as the nicotine concentration increased. The most noticeable change occurred at the 10-min mark after administering the greatest concentration of nicotine (0.63 mg/kg), as seen in previous studies [19,20]. 

To further validate our previous findings and test the efficacy of our CMOS device, we introduced a microdialysis system in tandem with our microimaging platform to obtain a more complete perspective of nicotine’s effect on dopaminergic stimulation with regard to the mesolimbic pathway by taking advantage of nicotine’s ability to increase DA release [15]. We concluded that the fluorescence intensity recorded in the VTA following stimulation of the DA neurons could be observed via DA output in the NAc, consistent with similar findings using optogenetic techniques [21]. Recently, this experimental platform was adapted to investigate nociception by enabling the visualization of serotonin. In tandem with microdialysis, our group observed brain activity following a noxious stimulus induced by formalin injection in mice’s hind paws [16]. The DRN is a group of neurons in the midbrain responsible for the synthetization and release of serotonin [22,23]. The CeA and the ACC are thought to play critical roles in regulating pain via serotonin, with the CeA regulating fear and anxiety and the ACC regulating one’s interpretation of acute and chronic pain [24,25]. We observed that as the fluorescence intensity increased, the serotonin concentration recorded from the microdialysis probes implanted in the CeA and AAC also increased. Our group successfully demonstrated that the micro-CMOS imaging device platform can monitor a range of neurotransmitters to investigate different neurological pathways as well as different drug effects. 

### 3.3. Visualization and Analysis of Drug Administration

Opioids are a pervasive class of drug, widely utilized in medical settings and frequently abused in illicit contexts, and their effects prove potent for pain relief and addictions, often inducing a sedative state [10,26]. Morphine is one of the most common opioids for pain relief, and as a result, it is one of the most frequently abused prescription drugs [27,28]. The central nervous system (CNS) is responsible for proliferating the effects of opioids, resulting from the modulation of μ-opioid receptors on GABAergic interneurons within the VTA [29,30]. The GABAergic neurons that regulate DA are inhibited, leading to the unattenuated production of DA in the VTA and the increased deposition of DA in the NAc [31,32]. Since studies conclude morphine inhibits GABAergic neuronal activity, leading to the uninhibited excitation of DA neurons, we expect an extended fluorescence intensity fluctuation [33]. Our results suggest reaffirmation of these studies due to the characteristic trend observed following subcutaneous injection of morphine to adult mice. When visualized as an image from the CMOS sensor given at a 5-min average, we observed a steady increase in fluorescence intensity across the entirety of the imaging area, though a gradual decrease began near the 110-min average image. The slope of the decrease in intensity was observed to be relatively gradual. It can be inferred that the biochemical mechanism resulting from morphine exposure caused the gradual change in fluorescence. As GABA neurons are inhibited, the attenuation of excited DA neurons cannot occur, causing DA release from an excitatory state for an extended period. 

Using our unique analysis software, we are able to generate ROIs across the CMOS imaging field, allowing us to pinpoint specific regions of intense or stable activity. The Python-based program detects slight changes during each frame of the recording and identifies each area that presents heightened local activity. It achieves this by taking advantage of adaptive binarization and morphological transformations. When identifying a specific ROI from the CMOS imaging area and adapting it to graphical output, we can observe more clearly the fluorescence fluctuation characteristics specific to the relationship between morphine and the mesocorticolimbic system—an extended increase in fluorescence intensity with a gradual decline below baseline. Our recent studies support the suggestion that the change in fluorescence intensity is directly proportional to the presence of extracellular DA in the NAc. Therefore, we can infer that the extracellular DA initially dumped in the NAc during peak neuronal activation time within the first 90 min is due to the inhibition of GABAergic neurons in the VTA, and the steady decline over time in fluorescence intensity is potentially due to the metabolite-mediated removal of extracellular DA in combination with the reactivation of GABAergic interneurons [34]. 

Another important class of drug often related to abuse and addiction, especially in an illicit context, is stimulants; stimulants are equally as germane to the discussion of DA dysregulation, and the resulting consequences following habitual usage due to their radical effect on the CNS, which differs from effects derived from opioid usage, are critical to investigate. While opioids bind to the μ-opioid receptors of GABAergic neurons, stimulants directly increase the release of specific neurotransmitters or inhibit their reuptake [35]. Cocaine is an illegal stimulant frequently used by those addicted to drugs. The DAT, located in the neuron terminals of dopaminergic neurons, is a membrane transporter protein responsible for the reuptake of DA molecules from the synapse back into the pre-synaptic space [36]. Cocaine blocks the DAT, inhibiting the reuptake mechanism and leading to a rapid and concentrated buildup of DA in the synaptic cleft, and it increases the DA neurotransmissions due to the lack of reuptake feedback [37]. This mechanism is crucial because it allows for more intense interactions with post-synaptic receptors. 

The CMOS imaging area captures the fluorescence activity of nearly the entirety of the NAc. When analyzing the 5-min image averages during cocaine experimentation, it was clear that the administration of cocaine incited a front-loaded dopaminergic event with a steep drop-off beginning at the 120-min mark. Additionally, the fluorescence intensity continued to drop well below the baseline value. This phenomenon may indicate an overcompensation reaction from GABAergic neurons within the VTA due to the extended period during which a lack of reuptake feedback occurs and may be comparable to the “comedown phase” experienced by humans as the effects of the cocaine wears off, during which DA levels plummet [38]. When assessing a single ROI from the imaging field, the characteristic trend following cocaine administration becomes more apparent. The sharp decline in fluorescence intensity occurs after 120 min, becoming stable below the baseline after some time. This stability further implies that the dopaminergic system is heavily overregulating DA production. To attest to these findings, a control group was observed, as seen in Figure 6. It was noted that negligible change in fluorescence occurred during the 90 min following the saline injection, allowing us to infer that the modulation of DA witnessed from the drugs is indeed pharmacologically induced.

**Figure 8:**
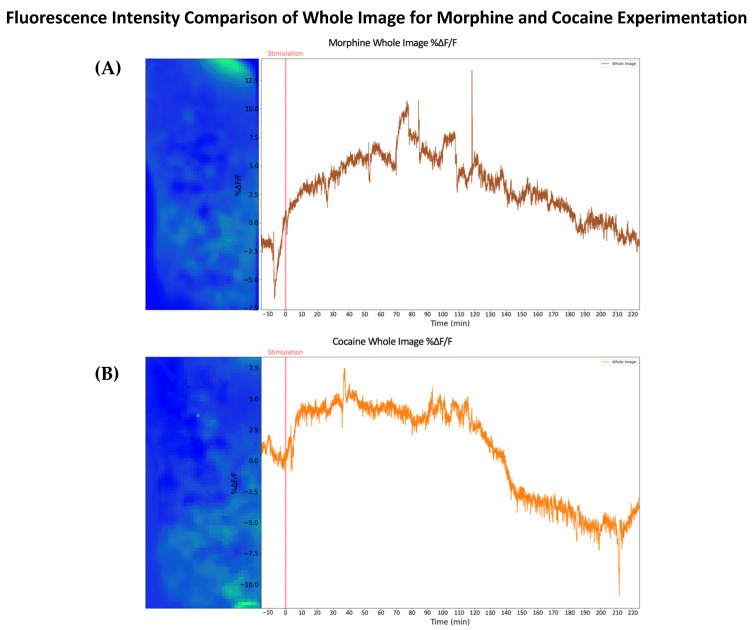
Fluorescence intensity changes (*ΔF/F*_0_) in the entire CMOS imaging area in the NAc of representative adult mice following either a subcutaneous injection of morphine or cocaine. An identical experimental protocol was implemented for both animals. (**A**) Whole image analysis of the fluorescence intensity changes during the morphine experiment. (**B**) Whole image analysis of the fluorescence intensity changes during the cocaine experiment.

Our results suggest we are able to observe that morphine and cocaine administration effectively modulates the production of DA and the resultant extracellular DA concentration in the NAc, and the associated change in fluorescence intensity trends differs between each drug. It can be inferred that this is due to the different biochemical mechanisms responsible for the modulation of the DA pathways, which is further highlighted when we evaluate the change in fluorescence of the entire imaging field instead of a single ROI, as shown in Figure 8. Characteristics of the DA release from the morphine-administered animal in Figure 8A take on a gradual increase and decrease in intensity compared to the cocaine-administered animal in Figure 8B. Furthermore, our ability to observe fluorescence proportional to extracellular DA in the NAc suggests that our previous microdialysis findings from both optogenetic and nicotinic stimulation in previous studies were, in fact, due to the respective stimuli and corroborate our current findings [15,19]. Finally, our statistical analysis of each experimental group shows significant modulation following drug administration. On average, morphine administration produced a more gradual fluctuation in fluorescence intensity than the cocaine experimental group. As expected, the stimulant produced a faster and more powerful fluctuation of DA, demonstrating that tracking the release of the DA molecule, and potentially other neurotransmitters, in the deep brain in real time using our CMOS imaging platform is feasible.

## 4. Materials and Methods

### 4.1. Ethics Statement and Animal Care

All experimental protocols and surgical procedures were approved by the Animal Care Committee of the Graduate School of Pharmaceutical Sciences of Nagoya City University and conducted with respect to the guidelines of the National Institute of Health and the Japanese Pharmacological Society (approval number: H25-P-01). This study included male C57BL/6 mice (6 weeks old; CLEA Japan, Shizuoka, Japan) (*n* = 9). All mice were housed in a room maintained at 23 ± 2 °C with an alternating 12-hour light-dark cycle and had food and water ad libitum. 

### 4.2. Implantable Imaging Device and Fabrication

**Figure 9:**
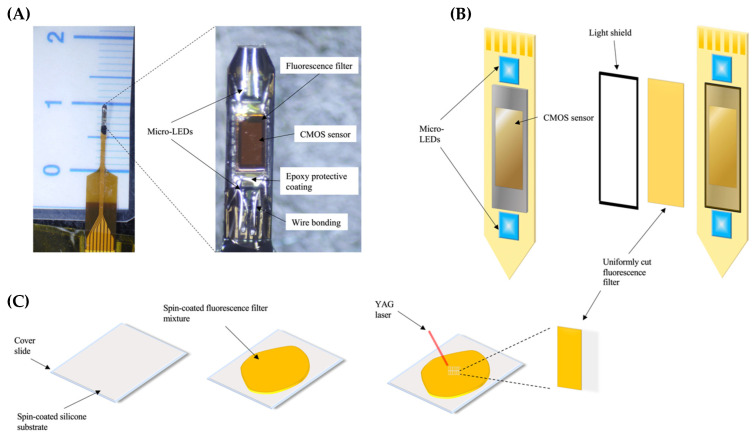
CMOS design and filter fabrication process. (**A**) On the left, the custom-fabricated CMOS imaging device is pictured. The CMOS chip is 450 µm × 1275 µm, and the imaging area is 300 µm × 675 µm in size. To the right, the structure of the device is highlighted. (**B**) Fabrication of the imaging device is demonstrated. Initially, the µ-LEDs and CMOS chip are adhered to the flexible substrate. The custom-made filter is adhered to the imaging surface. A black-light shield and epoxy are introduced for erroneous light absorption and hardware protection. (**C**) The fluorescence absorption filter fabrication process is illustrated. Spin-coating is used on a glass slide to create a smooth silicone substrate layer. The fluorescence filter mixture is spin-coated on the silicone substrate and cured. YAG laser technology is implemented to produce identically uniformly cut filters.

The CMOS imaging device was customized and designed to investigate neural pathways within the rodent brain, as seen in Figure 9. To enable access to deep brain regions, the device was fabricated in a needle shape using an FBC substrate (Taiyo Industrial Co. Ltd., Wakayama, Japan), with the CMOS sensor (TSMC, Hsinchu, Taiwan) and micro-LEDs (ES-VEBCM12A, Epistar Corporation, Hsinchu, Taiwan) integrated at the tip of the device. The LEDs (280 μm × 305 μm) integrated into the device, which emit a wavelength of 473 nm, were used to excite the previously injected dLight1.2 AAV (Addgene, Watertown, MA, USA), enabling the visualization of DA release into the NAc. We arranged the pixels of the CMOS imaging sensor in a rectangular fashion, consisting of 40 × 90 pixels, with each pixel being 7.5 μm by 7.5 μm in length. By arranging the pixels in this fashion, we eliminated the traditionally difficult task of observing deep brain regions with a vertical perspective, allowing visualization of the entirety of the NAc. 

Fabrication of the fluorescence imaging device begins with constructing the fluorescence filter. Covering the surface of the CMOS sensor, light emitted by the micro-LEDs is blocked by the filter to reduce erroneous fluorescence, allowing only emission light from the dLight1.2 expressing neurons to be observed. We used a spin-coating technique to ensure uniformity when making the custom filters. A cover glass (24 × 24) is first spin-coated with a silicone substrate (Norland Products, Jamesburg, NJ, USA) and allowed to cure overnight, acting as a base for the filter fabrication process. To create the filter, Valifast yellow 3150 dye (Orient Chemical Industries, Osaka, Japan), cyclopentanone (FUJIFILM Wako Pure Chemical Co., Osaka, Japan), and NOA63 resin (Norland Products) were combined using a 1:1:1 ratio based on weight. After being placed under a vacuum and allowed to rest overnight, the filter was spin-coated over the silicon substrate. The filter was soft-cured by baking for 10 min at 100 °C. We then cut the filters, matching the exact size of the sensor using a yttrium aluminum garnet (YAG) laser (Callisto VL-C30RS-GV, TNS Systems LLC, Matsudo, Japan). We then adhered the CMOS sensor and micro-LEDs to the FPC to maintain uniformity; using clear epoxy resin (Z-1; Nissin resin, Yokohama, Japan), the CMOS sensor was attached to the substrate surface, with micro-LEDs placed directly to the top and bottom of the sensor, and baked it for 10 min at 120 °C to set the epoxy. Wire bonding (7400C-79, West Bond, Anaheim, CA, USA) was then performed using aluminum wire (TANAKA Holding Co., Ltd., Tokyo, Japan) to connect the IO pads of the CMOS sensor and micro-LEDs to the integrated circuitry of the FPC substrate. A filter was then placed on the sensor, covering the entirety of the imaging field, and hard-cured by heating under vacuum for 120 min at 120 °C. We introduced a light shield to reduce the excitation light leakage from the micro-LEDs. The light shield consisted of a mixed solution incorporating blue (SR-3000L), green (SB-3000L), and red resists (SG-3000L, FUJIFILM Electronic Materials Co., Ltd., Kanagawa, Japan) using a 1:1:1 ratio based on weight. The light shield was applied to the side surfaces of the CMOS sensor and micro-LEDs and baked for 10 min at 120 °C. Epoxy resin was applied to the micro-LEDs and the aluminum wires, covering them completely to protect the components from damage, and was baked for 10 min at 120 °C. The device was then coated in a thin layer of Parylene-C (5 g of dichloro-c-cyclophane) to ensure biocompatibility and water resistance, which was applied using a Parylene coating chamber (PDS 2010, Specialty Coating Systems, Indianapolis, IN, USA). With approximately 3µm thickness, the coating protects without inhibiting flexibility.

### 4.3. Stereotaxis Surgery

Each mouse received two surgeries: (i) dLight1.2 AAV injection and (ii) CMOS imaging device implantation. The general surgical procedures for AAV injection and CMOS imaging device implantation are detailed in our previous studies [14,15,16,19].

### 4.4. dLight1.2 AAV Injection

Adult mice underwent AAV injection surgery. We injected AAV5-hSyn-dLight1.2 (#111068-AAV5; Addgene, Watertown, MA, USA) into the NAc to achieve virus-mediated expression of dLight1.2, a genetically encoded optical DA sensor capable of enabling the fluorescence detection of DA released into the NAc. The virus was injected at 0.15 µL/min for 5 min, requiring a minimum of 14 days for full expression to develop.

Each mouse was anesthetized using a pentobarbital solution administered via an IP injection. After ensuring the animal was unconscious, we fixed the mouse with its head in a stereotaxic surgery station (Narishige, Tokyo, Japan). An incision was created along the sagittal suture to expose the skull, and the surface of the skull was cleaned of tissue. Bregma was identified, the position for the burr hole was marked, and the height of bregma and lambda were adjusted to be equal. We then made a burr hole above the NAc, and the dura was broken. The injection was administered at the following coordinates (calculated via literature): anteroposterior (AP): +1.5 mm, mediolateral (ML): +0.9 mm, and dorsoventral (DV): −4.6 mm. A capillary glass needle loaded with the dLight1.2 AAV was slowly inserted into the previously mentioned location, and the injection ensued. Following the completion of the injection, the needle remained for an additional 5 min to ensure complete expulsion of the virus. Carefully, the needle was removed and discarded appropriately. The incision was closed using a suture, and the animal was monitored until it was fully awake. At this point, the animal was returned to its cage and post-operatively monitored for the following weeks.

### 4.5. CMOS Imaging Device Implantation

Two weeks following the AAV injection, the implantation of the CMOS imaging device in the NAc was performed, with the implantation located at the exact coordinates of the AAV injection. The animal was anesthetized using the recommended dose of pentobarbital, administered through IP injection. After ensuring complete anesthetization, the mouse’s head was fixed in a stereotaxic surgical station and prepared for surgery. The scalp hair was removed, and an incision atop the sagittal suture was made, revealing the skull. The cranial surface was thoroughly cleaned of bodily fluids and connective tissues to prevent the dental cement from detaching from the skull post-operatively. Bregma was identified and marked, and the height of bregma was confirmed to be equal to lambda, ensuring the proper angle of insertion of the imaging device. We drilled a burr hole above the NAc, and three additional burr holes were created around the insertion site for screw placement. These stainless steel screws were subsequently implanted to maintain stability and reduce the risk of device detachment. The device was initially placed into the brain so that the bottom of the sensor was at the same level as the brain’s surface to ensure accuracy regarding the implantation location. We then inserted the device slowly to confirm that no bending of the device occurred, reducing further damage to the brain tissue. After reaching the appropriate location, we applied dental cement incorporated with powdered carbon (Sigma-Aldrich, St. Louis, MO, USA) around the device and the surrounding screws. Since the CMOS sensor is sensitive to extraneous light, implementing carbon powder further inhibits nonfluorescent light from reaching the sensor by providing the dental cement with a dark tint. The incision was then sutured around the implantation, and the animal was monitored until fully conscious. Parafilm was tightly wrapped around the connector side to prevent damage to the imaging device, and each mouse was placed separately into their respective home cage. 

### 4.6. Experimental Setup and Fluorescence Imaging of the NAc

**Figure 10:**
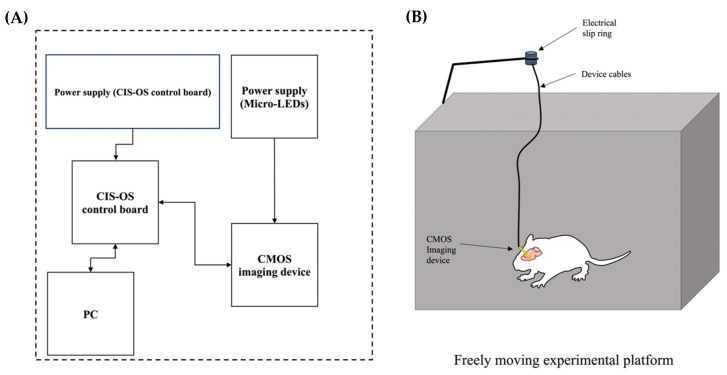
The experimental setup incorporates the newest CIS-OS analysis hardware and software version. (**A**) The feedback system of the experimental design is diagrammed. The custom CIS-OS control board communicates with the implanted CMOS imaging device to present real-time imaging on the connected PC. (**B**) A freely moving experimental platform was implemented to establish realistic experimental conditions, providing practical experimental results. The electrical slip ring prevented the twisting and entanglement of wires, and the CMOS device’s minimally invasive design allowed for full range of motion.

To investigate DA release concerning the mesolimbic pathway, we introduced adult mice to pharmacological stimulation using morphine, cocaine, and control and recorded the DA output to the NAc in a freely moving environment (*n* = 9), as seen in Figure 10. Following the CMOS imaging device implantation surgery, the mice were allowed two weeks to recover. Unlike our previous studies, which provided only 24–72 h of recuperation, waiting 14 days allows for a more complete recovery for the animal. Therefore, we reduced the risk of potential side effects from the surgery, and the brain tissue surrounding the implantation had adequate time to readjust. 

Each mouse underwent experimentation separately and received a single subcutaneous injection per experiment. The experimental setup was established using an enclosure that facilitated freely moving conditions. Our novel, custom CIS-OS platform was integrated into the study to enable successful visualization of the imaging area. Unlike our previous CIS-NAIST CMOS imaging platform, this version allows for streamlined recording with a smaller, more easily portable control board accessible via ethernet, a program with a more simplified user interface, and easier access to hardware troubleshooting using integrated testing tools. The custom connector that is attached to the implanted device uses only six wires: four wires power the CMOS sensor and deliver information to the CIS-OS control board, and the remaining two wires enable fluorescence activation by providing power to the device-integrated micro-LEDs using a current generator. An additional current generator is used to power the CIS-OS control board, and the control board sends information to the program via an ethernet connection. Overall, the experimental setup allows for deep brain investigation and mapping of notoriously difficult-to-reach locations without significantly compromising animal movement and the resulting naturally occurring behavior.

Following the preparation of the experimental setup, the connector was attached to the implanted device, and we placed the mouse in the freely moving enclosure. The device was powered on, and proper function was ensured. Being that all other lights were extinguished at this point, blue lights surrounded the enclosure to maintain the visibility of the animal during the experiment. Blue lights were chosen because they produce negligible interference for recordings due to the yellow fluorescence filter covering the CMOS imaging surface. A background recording was obtained. The LEDs on the device were then powered, and the recording was started, with fluorescence being captured at ten frames per second (FPS). The mouse was recorded for two hours before injection, acting as a control. At the two-hour mark, the mouse was administered morphine via subcutaneous injection and then released back into the enclosure. The mouse was then monitored post-stimulation. 

### 4.7. Imaging Analysis and ROI Analysis Program

Our newest imaging software and platform, CIS-OS 1.0, enabled fluorescence imaging using the custom CMOS imaging device. Each experimental session was visualized six hours after injection, with reference taken before injection. Each pixel was normalized to the baseline average fluorescence value. To find our percentage change in fluorescence intensity, the mathematical equation was applied:

$$\frac{∆F}{F_{0}}\%=\frac{F-F_{0}}{F_{0}}\times 100$$

where *F* is the fluorescence intensity and $F_{0}$ is the baseline fluorescence. As utilized in one of our papers, regions of interest (ROIs) were identified by applying an algorithm based on Python, composed of adaptive binarization and morphological transformations, further detailed in our previous study [16]. 

### 4.8. Implantation Site Confirmation

Following each animal’s final experiment, each mouse underwent a terminal procedure to clean and fix the brain as preparation for slicing. Perfusion fixation was performed by sedating the animal and opening the thoracic cavity to expose the heart. A needle was inserted into the right atrium, and PBS was pumped throughout the cardiovascular system. The aorta was dissected and severed to allow for the removal of the blood. Following PBS treatment, 4% paraformaldehyde (PFA, Thermo Fisher Scientific, Waltham, MA, USA) was propagated throughout the cardiovascular system to fix the brain. After treatment, the brain was removed and stored in a glucose solution. The brain was sliced using a VT1200 semiautomatic vibrating blade vibratome (Leica, Deer Park, IL, USA), with each slice having a thickness of 100 µm. The slices were then placed on slides and analyzed using fluorescence microscopy.

### 4.9. Statistical Analysis

Statistical significance was determined using one-way repeated measure analysis of variance (ANOVA) regarding the fluorescence ratios and their respective time points. A *p*-value of 0.05 was considered statistically significant, and all data are represented as mean ± standard error.

## 5. Conclusions

Previous studies demonstrated that our novel deep brain imaging platform could study nociceptive pathways via formalin injection and optogenetically induced and physiologically induced stimulation of addiction pathways using fluorescence imaging captured by micro-CMOS technology in real time. This study shows that our CMOS device experimental setup can also be used to investigate the effects of pharmacologically induced stimulation regarding the mesolimbic pathway by identifying the DA transmitter rather than dopaminergic neuronal firing. We demonstrate a relationship between the administration of morphine and cocaine and the modulation of DA output to the NAc using fluorescent imaging facilitated by dLight1.2 expression. Morphine administration incited a gradual DA increase and decrease within the NAc, while cocaine introduced a steeper fluctuation in DA modulation. It further verifies the biochemical mechanisms responsible for these fluctuations following morphine or cocaine exposure. Analysis of the successful stimulation and recording of the NAc allows us to validate further our previous microdialysis findings concerning DA concentration increase in the NAC following stimulation of both rats and mice. Finally, our results suggest that our CMOS imaging platform can be used to track neurotransmitters other than DA, expanding the possibilities for deep brain neural mapping.

## Data Availability

The data presented in this study are available on request from the corresponding author.

## Abbreviations

DA	Dopamine
VTA	Ventral tegmental area
NAc	Nucleus accumbens
GABA	γ-aminobutyric acid
DAT	Dopamine transporter
PFC	Prefrontal cortex
CMOS	Complementary metal-oxide semiconductor
FPC	Flexible printed circuit
AAV	Adeno-associated virus
ROI	Region of interest
CNS	Central nervous system
GECI	Genetically encoded calcium indicator
